# Diversity of Gene Clusters for Polyketide and Nonribosomal Peptide Biosynthesis Revealed by Metagenomic Analysis of the Yellow Sea Sediment

**DOI:** 10.3389/fmicb.2018.00295

**Published:** 2018-02-27

**Authors:** Yongjun Wei, Lei Zhang, Zhihua Zhou, Xing Yan

**Affiliations:** Key Laboratory of Synthetic Biology, Institute of Plant Physiology and Ecology, Shanghai Institutes for Biological Sciences, Chinese Academy of Sciences (CAS), Shanghai, China

**Keywords:** PKS-I and NRPS diversity, gene cluster, biosynthesis, metagenomics, marine sediment

## Abstract

Polyketides (PKs) and nonribosomal peptides (NRPs) are widely applied as drugs in use today, and one potential source for novel PKs and NRPs is the marine sediment microbes. However, the diversities of microbes and their PKs and NRPs biosynthetic genes in the marine sediment are rarely reported. In this study, 16S rRNA gene fragments of the Yellow Sea sediment were analyzed, demonstrating that *Proteobacteria* and *Bacteroidetes* accounted for 62% of all the bacterial species and *Actinobacteria* bacteria which were seen as the typical PKs and NRPs producers only accounted for 0.82% of all the bacterial species. At the same time, PKs and NRPs diversities were evaluated based on the diversity of gene fragments of type I polyketide synthase (PKS) ketosynthase domain (KS), nonribosomal peptide synthetase (NRPS) adenylation domain (AD), and dTDP-glucose-4,6-dehydratase (dTGD). The results showed that AD genes and dTGD genes were abundant and some of them had less than 50% identities with known ones; By contrast, only few KS genes were identified and most of them had more than 60% identities with known KS genes. Moreover, one 70,000-fosmid clone library was further constructed to screen for fosmid clones harboring PKS or NRPS gene clusters of the Yellow Sea sediment. Nine selected fosmid clones harboring KS or AD were sequenced, and three of the clones were assigned to *Proteobacteria*. Though only few *Actinobacteria* 16S rRNA gene sequences were detected in the microbial community, five of the screened fosmid clones were assigned to *Actinobacteria*. Further assembly of the 9 fosmid clones resulted in 11 contigs harboring PKS, NRPS or hybrid NPRS-PKS gene clusters. These gene clusters showed less than 60% identities with the known ones and might synthesize novel natural products. Taken together, we revealed the diversity of microbes in the Yellow Sea sediments and found that most of the microbes were uncultured. Besides, evaluation of PKS and NRPS biosynthetic gene clusters suggested that the marine sediment might have the ability to synthesize novel natural products and more NRPS gene clusters than PKS gene clusters distributed in this environment.

## Introduction

Natural products of polyketides (PKs) and nonribosomal peptides (NRPs) are secondary metabolites of microbes, which can help microbes adapt to environment and resist to stressful natural conditions. Until now, more than 23,000 natural products of PKs and NRPs had been identified and characterized, and they had been widely use as antibiotic and antitumor medicine (Bérdy, 2005; Walsh, 2007; Demain and Sanchez, 2009; Newman and Cragg, 2012; Katz and Baltz, 2016). Additionally, many previously identified PKs and NRPs were recovered from isolated bacteria of terrestrial environments (Handelsman et al., 1998; Daniel, 2004), especially from the phylum of *Actinobacteria* (Bérdy, 2005; Fenical and Jensen, 2006; Bull and Stach, 2007). However, nowadays, PKs and NRPs recovered from easily cultured microbes are often proven to be the same ones previously identified, suggesting the possibility of getting novel PKs and NRPs by traditional cultivation-dependent method dramatically decreases (Tulp and Bohlin, 2005; Xiong et al., 2015).

The oceans, which cover more than 70% of the earth, represent a rich source of valuable novel natural products (Molinski et al., 2009). More than 1,000 novel compounds had been identified from the ocean in the past few years (Blunt et al., 2013, 2014, 2015). It was estimated that *Actinobacteria* was the main source of bioactive PKs and NRPs in the ocean (Fiedler et al., 2005; Jensen et al., 2005). 16S rRNA analysis showed that *Actinobacteria* bacteria were not the most abundant phyla in the marine sediment, suggesting there might be other potential PKs and NRPs producer which can produce novel PKs and NRPs (Zhu et al., 2013). Moreover, 99% of the bacteria were recalcitrant to cultivate, indicating that uncultured marine microbes might be the potential reservoir to discover novel natural products (Torsvik et al., 1996; Whitman et al., 1998; Rappé and Giovannoni, 2003). Besides, different natural product biosynthetic genes are distributed in phylogenetic similar microbial communities (Reddy et al., 2012), showing the unrevealed marine environments are the attractive starting points to recover novel bioactive compounds (Brady et al., 2002; Piel, 2011; Wilson and Piel, 2013).

PKs are mainly synthesized by type I polyketide synthase (PKS-I) and type II polyketide synthase (PKS-II) gene clusters, and gene fragment of PKS-I ketosynthase (KS) domain and PKS-II KSα domain are often used for PKs diversity evaluation. In the meanwhile, NRPs are synthesized by nonribosomal peptide synthetase (NRPS), and NPRS adenylation (AD) domain is used for NRPs diversity evaluation (Reddy et al., 2012). Nowadays, culture-independent metagenomic methods have successfully used for discovering of novel natural product biosynthetic gene clusters from diverse environments, and can be used to evaluate natural product biosynthetic gene diversity in the marine sediment (Brady et al., 2009; Wilson and Piel, 2013). Deep sequencing of NRPS and PKS gene fragments in the marine sponges confirmed that only a small amount of the recovered genes assigned to *Actinobacteria*, suggesting that PKs and NRPs recovered from the marine environment would be different from previously known ones mainly produced by *Actinobacteria* (Woodhouse et al., 2013). Moreover, the dTDP-glucose-4,6-dehydratase (dTGD) which can glycosylate natural products by 6-deoxyhexose (6DOH) are usually parts of natural product biosynthetic gene clusters and involved in secondary metabolism, and can be used to evaluate 6DOH-modified NRPs and PKs diversities in the environments (Thibodeaux et al., 2007; Bruender et al., 2010; Chen et al., 2011).

In this study, gene fragments of 16S rRNA, NRPS AD domain, PKS-I KS domain and dTGD were sequenced to evaluate bacteria and their natural product biosynthetic gene diversities in the Yellow Sea sediment. In order to recover natural product biosynthetic genes, one fosmid clone library were constructed using same DNA extracted from the Yellow Sea sediment. Nine selected fosmid clones harboring natural product biosynthetic genes were recovered and their natural product biosynthetic gene clusters were analyzed.

## Materials and methods

### Sample collection, DNA isolation and the fosmid clone library construction

The marine sediment samples were collected at the depth of 50–100 m in the summer of 2010 from several adjacent sites of undisturbed environment in the Yellow Sea sediment close to Rizhao, Shangdong province, China (Xiong et al., 2015). They were stored in sterilized plastic bags after being taken from the deep sea and transported to the laboratory at 4°C. As there might be some hazard microbes in the marine samples, the extraction experiment was carried out according to safety procedures of our institute. The sediment samples were mixed together, and total DNA was extracted from mixed sediment samples by a previously described protocol (Zhou et al., 1996; Geng et al., 2012). 10 ml PBS buffer was added to the mixed sediment samples and large particulates were removed by briefly centrifuging at 200 × g for 5 min. Cell pellets were obtained by further centrifugation (9,000 × g, 5 min), and 7 ml extraction buffer (100 mM Tris-HCl [pH 8.0], 100 mM EDTA [pH 8.0], 1.5 M NaCl, 100 mM Na_3_PO_4_,1% cetyl trimethyl-ammonium bromide [wt/vol], 1% sodium dodecyl sulfate [wt/vol], 1 mg/ml proteinase K) were added to suspend the cell pellets. The suspension was incubated at 55°C for 20 min and then 70°C for 10 min. The crude lysate was centrifuged at 17,000 × g for 10 min, and the cellular debris was pelleted. The supernatant of the crude lysate was moved to new centrifuge tubes and extracted with phenol/chloroform/isoamyl alcohol (25:24:1) for twice, followed by extraction with chloroform/isoamyl alcohol (24:1) for one time. The crude DNA was precipitated from the supernatant with the addition of 0.6 volumes of isopropyl alcohol and collected by centrifugation (5,400 × g, 10 min). The pelleted DNA was washed with 70% ethanol and resuspended with 200 μl TE buffer (10 mM Tris, 1 mM EDTA [pH 8.0]) containing 10 μg ribonuclease solution (RNase).

The high molecular-weight DNA (about 40 kb) was separated by CHEF electrophessis (Bio-rad, USA) and electroeluted from the agarose. Some DNA was blunt ended, ligated into the fosmid vectors, packaged into lambda phage and transfected into *Escherichia coli* EPI300 to construct a fosmid clone library according to the Copy-Control™ HTP Fosmid Library Production Kit manual (Epicentre, San Diego, CA, USA). The fosmid clone library contained more than 70,000 clones and all of them were preserved at 384-well plates. Besides, some DNA was used to amplify conserved domains of the natural product biosynthetic gene fragments and 16S rRNA gene fragments.

### PCR amplification of gene fragments from marine sediment samples

Primers designed to recognize conserved regions in PKS-I ketosynthase (KS), NRPS adenylation (AD), PKS-II ketosynthase alpha (KSα) and dTGD genes were selected to investigate the natural product biosynthetic gene diversities in the marine sediment (Table 1; Metsä-Ketelä et al., 1999; Du et al., 2004; Ginolhac et al., 2004; Ayuso-Sacido and Genilloud, 2005). The 16S rRNA gene V1-V3 regions were amplified with primers 27f/ P2 (Dong et al., 2011). For dTGD and 16S rRNA gene fragments, one adaptor (5′-CGTATCGCCTCCCTCGCGCCATCAG-3′) was added to the forward primer dTGDF and 27f (Parameswaran et al., 2007). To sequence different genes in one run and fetch the genes from the sequencing results, specific barcodes were added to each primer as described in Table 1.

**Table 1:** Primers used for amplification of conserved domains from natural product gene clusters.

**Candidate genes used for amplification**	**Predicted length (bp)**	**Primer name**	**Primer sequences (5′ → 3′)**	**Barcode**
Type I polyketide synthase KS domain fragments	700	KSLF	CCSCAGSAGCGCSTSYTSCTSGA	ACTCGTCT
		KSLR	GTSCCSGTSCCGTGSGYSTCSA	ACCTGTCT
Type II polyketide synthases KSα gene fragments	672	KSα-F	TSGCSTGCTTCGAYGCSATC	
		KSα-R	TGGAANCCGCCGAABCCGCT	
NRPS Adenylation domain fragments	795	A3	GCSTACSYSATSTACACSTCSGG	ATGCATGC
		A7R	SASGTCVCCSGTSCGGTAS	ATATATGC
dTDP-glucose-4,6-dehydratase gene fragment	530	dTGDF	GSGGSGSSGCSGGSTTCATSGG	TAGTAGTC
		dTGDR	GGGWRCTGGYRSGGSCCGTAGTTG	TATACATA
16S rRNA gene fragment	450	27f	AGATAAGAGTTTGATCMTGGCTCAG	ATCAGATC
		P2	ATTACCGCGGCTGCTGG	TGACACTA

For all the genes, LA Taq polymerase (Takara Biotechnology, Dalian) was used as the amplification polymerase and gradient annealing temperature was tried to amplify the corresponding gene fragments from the marine sediment. However, no PKS-II KSα sequences were amplified from the marine sediment. Another three primer pairs of K1(5′-TSAAGTCSAACATCGGBCA)/M6R(5′-CGCAGGTTSCSGTACCAGTA), (5′-CCSCAGSAGCGCSTSTTSCTSGA)/(5′-GTSCCSGTSCCGTGSGTSTCSA), and (5′-CCSCAGSAGCGCSTSCTSCTSGA)/(5′-GTSCCSGTSCCGTGSGCCTCSA) had been tried for KS gene amplification, but failed (No targeted gene fragment for K1/M6R, and very weaker fragments for the other two primer pairs) (Courtois et al., 2003; Ayuso-Sacido and Genilloud, 2005). The optimum 25 μl PCR reaction mixture used for gene amplification contained 5 ng marine sediment DNA, 0.5 μM each primer, 200 μM each deoxynucleoside triphosphate (dNTP), 1^*^GC Buffer I (Takara Biotechnology, Dalian) and 0.2 U LA Taq polymerase (Takara Biotechnology, Dalian). For KSLF/R and A3/A7R primers, a PCR protocol with an initial denaturation (5 min, 94°C), followed by 30 cycles consisting of 30 s at 94°C, 30 s at 59°C and 45 s at 72°C, and finally one cycle of 10 min at 72°C was used. For dTGDF/dTGDR and 27f/P2 primers, a PCR protocol with an initial denaturation (5 min, 94°C), followed by 25 cycles consisting of 30 s at 94°C, 30 s at 55°C and 45 s at 72°C, and finally one cycle of 10 min at 72°C was used.

### 454 Pyrosequencing and data processing of 16s rRNA and natural product biosynthetic gene fragments

PCR products of KS and AD gene fragments were gel purified using Axygen MinElute columns (Axygen Scientific Inc., USA) and equivalently mixed. Then two different 454-sequencing adaptors (Forward adaptor, 5′-CGTATCGCCTCCCTCGCGCCATCAG-3′ and reverse adaptor, 5′-CTATGCGCCTTGCCAGCCCGCTCAG-3′) were incorporated to the forward and reverse primers separately for parallel pyrosequencing by Roche 454 GS-FLX Titanium. For dTGD and 16S rRNA gene fragments, they were directly sequenced by Roche 454 GS-FLX Titanium. All the raw reads were deposited in the NCBI Sequence Read Archive (SRA) database under accession number of PRJNA403937.

Reads of these 4 gene fragments more than 400 bp were extracted by Acacia software with each barcode on the forward primers (Bragg et al., 2012). For KS and AD gene fragments, all the retrieved reads were trimmed to 400 bp from the forward primer site; for dTGD and 16S rRNA gene fragments, only reads with full-length (the reads should contain 27f/P2 or dTGDF/dTGDR primers) were selected for further analysis. Potential chimeric sequences of KS, AD and dTGD reads were filtered using Chimera filter in USEARCH with the commands of UCHIME denovo and ref (KS_REF, AD_REF and dTGD-REF, described below; Edgar, 2010; Edgar et al., 2011). Then KS reads were compared to the KS_REF databases, and any reads that did not align to a reference over a coverage of 90% and *E*-value of < 10e^−10^ were removed. For AD reads and dTGD reads were more divergent than KS reads, a cutoff *E*-value of 10e^−5^ was used in AD_REF and dTGD-REF databases searches. Finally, reads sorted by USEARCH were used for OTU classification (**Table 3**).

### Construction of reference sequence databases for KS, AD, and dTGD genes (KS_REF, AD_REF, and dTGD_REF)

Among all the databases collected information of the natural product biosynthetic genes, ClusterMine360 is an updated database which collects diverse microbial PKS and NRPS biosynthetic genes (Conway and Boddy, 2013). In order to generate a reference database of known AD and KS sequences, we downloaded all the gene clusters and domains from ClusterMine360 database. Then a primer-mapping search on all the domains was performed, only those sequences matched with KSLF/R or A3/A7R primers (allow for 3 mismatches) were selected and the sequences between the forward and reverse primers were retrieved. For all the sequences deposited in ClusterMine360 database were microbial PKS/NRPS biosynthetic genes, it was believed that the sequences retrieved from the database were authentic microbial KS and AD gene fragments. Though we used the same method to identify dTGD genes, only 7 sequences were retrieved from the ClusterMine360 database. To generate dTGD-REF with more dTGD gene sequences, another 51 reference functional dTGD sequences described before were retrieved from the NCBI database and added to the dTGD-REF (Chen et al., 2011). The KS_REF, AD_REF, and dTGD-REF databases contain 331 KS, 193 AD, and 58 dTGD gene sequences, respectively.

### 16s rRNA gene phylogenetic analysis

The retrieved full-length 16S rRNA gene reads (with forward and reverse primers) were processed with QIIME analysis and all the obtained 16S rRNA gene reads were classified into operational taxonomic units (OTUs) with uclust method at 97% similarity (Caporaso et al., 2010; Kuczynski et al., 2011). Then the chimeric sequences were identified with ChimeraSlayer program. After removing of chimeric sequences, all the 16S rRNA gene reads and a representative sequence of each OTU were used for the RDP-based phylum level classification, respectively. OTUs with more than 50 reads were considered as abundant OTUs and blasted for closest isolates from RDP database in this study.

### Assignment of natural product biosynthetic gene fragments to similarity-based OTUs

The retrieved reads of KS, AD, and dTGD reads were aligned with MAFFT alignments and clustered at appropriate identity into OTUs using USEARCH (Chen et al., 2011; Reddy et al., 2012). In order to construct phylogenetic tree, representative reads of OTU in each dataset, natural product biosynthetic gene fragments extracted from the contigs (describe below) and the corresponding sequences in the reference databases were aligned with MAFFT alignments. Circular phylogenetic trees of KS, AD, and dTGD genes were constructed using MEGA 5.2 with Neighbor-joining method and edited with FigTree v1.4 (http://tree.bio.ed.ac.uk/software/figtree/; Tamura et al., 2011). For AD genes, only AD OTUs contained more than 3 reads were used for AD tree construction.

Type I PKSs can typically be classified into two types, acyltransferase (AT)-less type I PKSs and canonical type I PKSs. In order to identify the evolutionary lineage of recovered KS gene fragments, the representative reads of each KS OTUs and 6 KS gene fragment identified from the fosmid contigs were translated into protein sequences using meta-prodigal (Hyatt et al., 2010). Another 12 reference KS gene sequences (9 AT-less type I PKSs KS gene fragments and 3 canonical type I PKSs KS gene fragments) were downloaded (Lohman et al., 2015). All the KS gene fragments were aligned with MAFFT alignments and used for phylogenetic tree construction using Maximum Likelihood method.

### Screening, sequencing and annotation of fosmid clones harboring natural product biosynthetic gene fragments

Besides investigating the diversity of natural product biosynthetic genes, the primers of KSLF/R and A3/A7R were used to screen fosmid clones harboring the natural product biosynthesis genes. The PCR mixture **(**20 μl) used for clone screening was composed with 0.2 μM dNTPs (Takara Biotechnology, Dalian), 0.5 μM of each primer, 5 ng DNA or 2 μl of overnight culture as template, 5% dimethyl sulfoxide (DMSO), 0.5 U rTaq polymerase and the recommend rTaq buffer (Takara Biotechnology, Dalian). The screening PCR protocol was initially with one cycle of 94°C for 5 min, followed by 40 cycles of 94°C for 30 s, 59°C for 30 s and 72°C for 1 min, finally with one cycle of 10 min at 72°C. Fosmid DNA extracted from each 384-well plate was used as template to screen out plates harboring natural product biosynthetic gene fragments. The overnight culture collected from each column or row of the positive plates were mixed and 2 μl of them was used as PCR template to screen for the positive fosmid clones harboring KS or AD domain. Then 8 fosmid clones contained AD genes and 1 fosmid contained KS genes were sequenced using 454 pyrosequencing and 12 contigs were assembled from these 9 fosmid clones using Newbler software version 2.6. Some KS domains and AD domains were extracted from the sequences by primer-mapping search of the sequences as described above and used for phylogenetic tree construction (allow 4 mismatches in each primer). The taxonomy of 12 contigs was determined with PhyloPythiaS analysis and annotated with fgenesb and BlastX (Patil et al., 2012). All the contigs were submitted to antiSMASH for further PKS and NRPS gene cluster annotation (Weber et al., 2015). The 12 contigs were deposited in Genbank and the accession numbers of them were MF964193-MF964204.

## Results

### Bacterial diversity in the marine sediment

**Figure 1:**
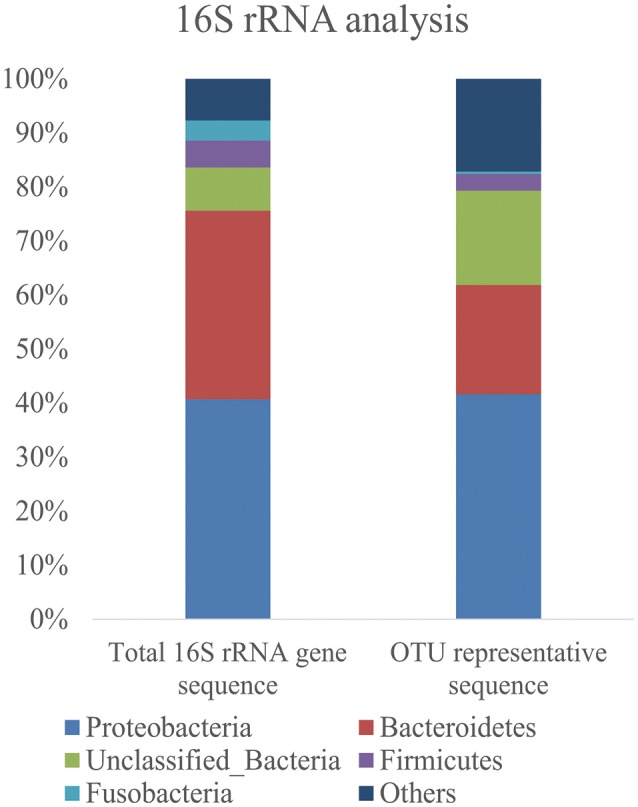
Bar graphs show the frequency of bacteria phyla identified from the Yellow Sea sediment samples. The distribution of total 16S rRNA gene sequences and representative sequences of each OTU was shown.

Bacterial diversity in the marine sediment was investigated using 16S rRNA gene fragments. A total of 7905 16S rRNA gene fragments were extracted from the original 454 pyrosequencing reads by Acacia software, and 6675 reads predicted to be full-length (with P2 and 27f primer in the reads) were used for further analysis. After removing of 100 chimera sequences, 6575 reads were classified into 1335 OTUs at 97% identity. The Good's coverage of the bacterial 16S rRNA genes was 89.08%, indicating that most bacteria of the marine sediment sample had been detected in this study. Based on analysis results of the 6575 16S rRNA reads and the representative reads of the 1355 OTUs, Proteobacteria were the most dominant phylum, which constituted 40.7% of the total 16S rRNA reads and 41.6% of all the representative OTU reads, respectively (Figure 1); followed by Bacteroidetes bacteria which composed 34.9% of total 16S rRNA reads and 20.2% of all the representative OTU reads, respectively. Only 0.3% of total 16S rRNA reads and 0.8% of the representative OTU were assigned as Actinobacteria bacteria which was believed to synthesize more than 45% of the known microbial PKs and NRPs in nature (Bérdy, 2005).

Of all the OTUs, 17 most abundant OTUs constituted 30.84% of the total 6575 16S rRNA gene reads (Table 2). Among them, OTU1242 constituted 8.29% and another 4 OTUs constituted more than 2% of the total reads. Interestingly, three of the 5 most abundant OTUs, OTU1242, OTU1041, and OTU1140, were assigned to Bacteroidetes, not assigned to the most abundant phylum of Proteobacteria in the sample. Most of the 17 abundant OTUs had less than 97% identity with their closest isolates, hinting that these abundant species in the marine sediment might be uncultured (Table 2). A total of 613 *Actinobacteria* stains had been isolated from the same marine sediment and 105 16S rRNA sequences had been released in previous study (Xiong et al., 2015). However, among all the 105 16S rRNA sequences, only three16S rRNA sequences (The Genbank accession numbers are JQ924069, JQ924085, and JQ924089, 100% identity of these 3 sequences) showed more than 95% identities with one OTU (containing 2 reads, and they showed 99.1 and 100% identities with these 3 sequences, respectively) identified in this study, suggesting that *Actinobacteria* bacteria identified by culture independent method were different from previously identified culturable *Actinobacrteria* in the same sample.

**Table 2:** The 17 most abundant OTUs in Yellow Sea sediment samples and their closest named isolates.

**OTU ID**	**Percentage (%)**	**Closest named isolates**	**Accession number**	**Identity (%)**
OTU1242	8.29	*Gillisia* sp. NP17	EU196340.1	96.52
OTU225	2.59	*Bizionia paragorgiae* str. KMM 6029	NR_025827.1	99.57
OTU1041	2.59	*Alviniconcha* sp. gill symbiont	AB189712.1	93.7
OTU1140	2.46	*Polaribacter* sp. str. UDC421	HM032000.1	96.54
OTU46	2.08	*Ilyobacter psychrophilus* str. FQ50	AJ877255.1	94.2
OTU1354	1.66	*Sulfurovum* sp. str. NBC37-1	AP009179.1	96.46
OTU1166	1.38	*Pelobacter venetianus*	U41562.1	97.33
OTU863	1.19	*Sphingomonas* sp. BAC84	EU131006.1	93.42
OTU232	1.17	*Propionigenium maris* str. DSL-1	Y16799.1	94.2
OTU828	1.16	*Sedimentibacter* sp. str. IMPC3	EF189918.1	81.98
OTU44	1.00	*Pelobacter venetianus*	U41562.1	96.89
OTU1246	1.00	*Geosporobacter subterrenus* str. VNs68	DQ643978.1	92.67
OTU872	0.96	*Pelobacter venetianus*	U41562.1	97.36
OTU472	0.90	*Oligobrachia mashikoi* endosymbiont D	AB271122.1	97.4
OTU469	0.87	*Brumimicrobium* sp. str. P99	EU195945.1	97.84
OTU15	0.79	*Alviniconcha* sp. gill symbiont	AB189712.1	95.49
OTU708	0.76	*Sulfurimonas autotrophica* str. OK5	AB088432.1	95.22

### Phylogenetic analyses of KS domain, AD domain, and dTGD gene fragments

After processing of the retrieved reads with Acacia software, 9995 KS reads, 4042 AD reads and 602 dTGD reads were obtained (Table 3). The KS and AD reads were classified into OTUs at 90% identity, and the dTGD reads were classified into OTUs at 80% similarity (Reddy et al., 2012). The KS, AD and dTGD reads can be classified into 27 OTUs, 1087 OTUs, and 279 OTUs at their selected identities, respectively (Table 3), showing NRPS and dTGD diversity might be more abundant than that of PKS in the microbial community of the marine sediment.

**Table 3:** Number of reads remained after each processing steps and number of OTUs classified based on selected identities.

	**PKS KS domains**	**NRPS AD domains**	**dTGD genes**
454 Reads fetched by Acacia	12,295	6,148	1,698
Reads >400 bp	10,943	4,899	1,221(full length)
No chimeras (de novo)	10,943	4,895	1,221
No chimeras (reference data)	10,943	4,895	1,221
Ref-homologs (BLAST)	9,995	4,042	602
OTUs	27 (90% identity)	1,087 (90% identity)	279 (80% identity)
Good's coverage	99.93%	85.75%	68.72%

**Figure 2:**
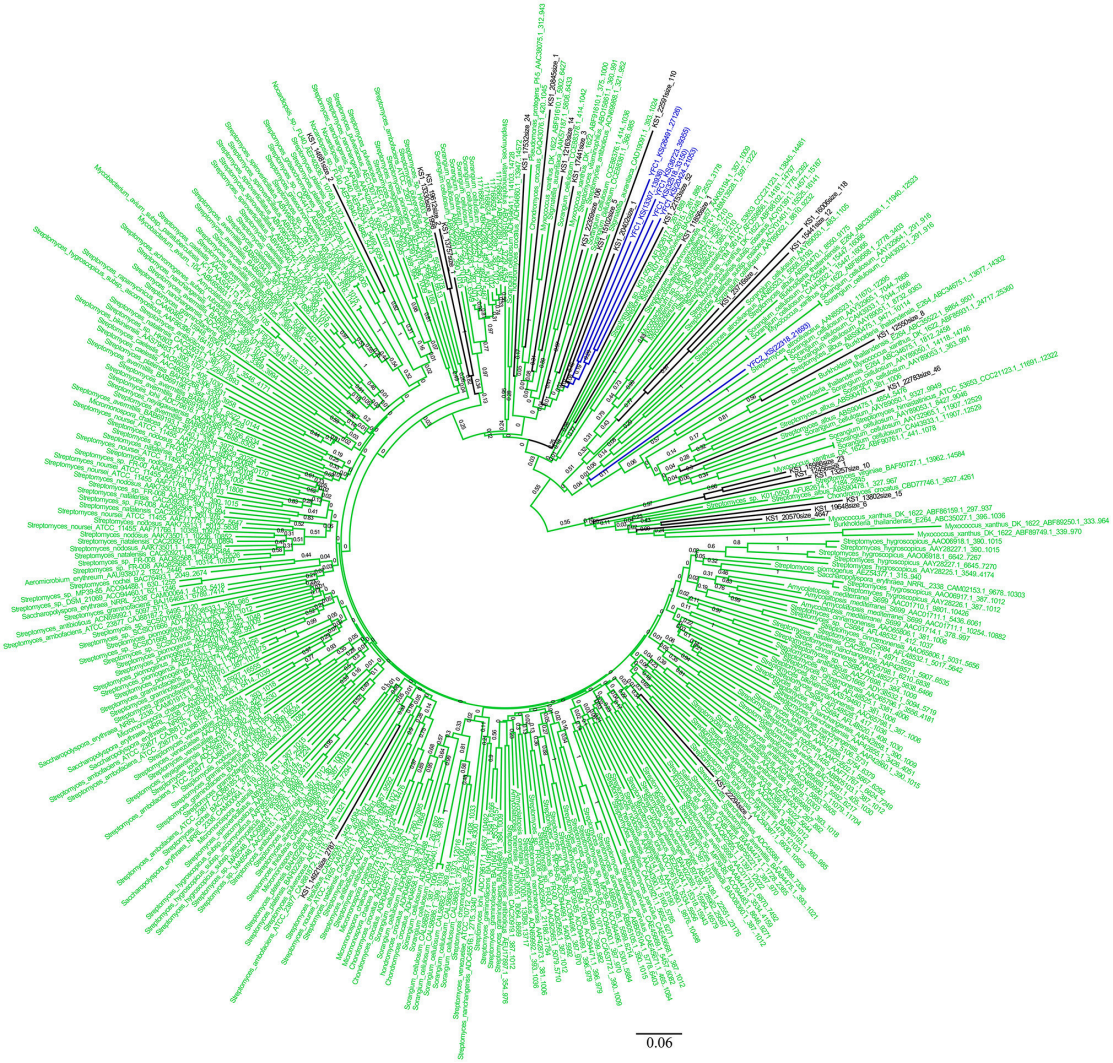
Phylogenetic tree of KS representative sequences of each OTU obtained from Yellow Sea sediment and the reference functional characterized KS sequences. All the representative sequences of each OTU clustered at 90% identities were marked with black. All the sequences identified in fosmid contigs were marked with blue. The functionally characterized KS retrieved from ClusterMine360 database were marked with green.

**Figure 3:**
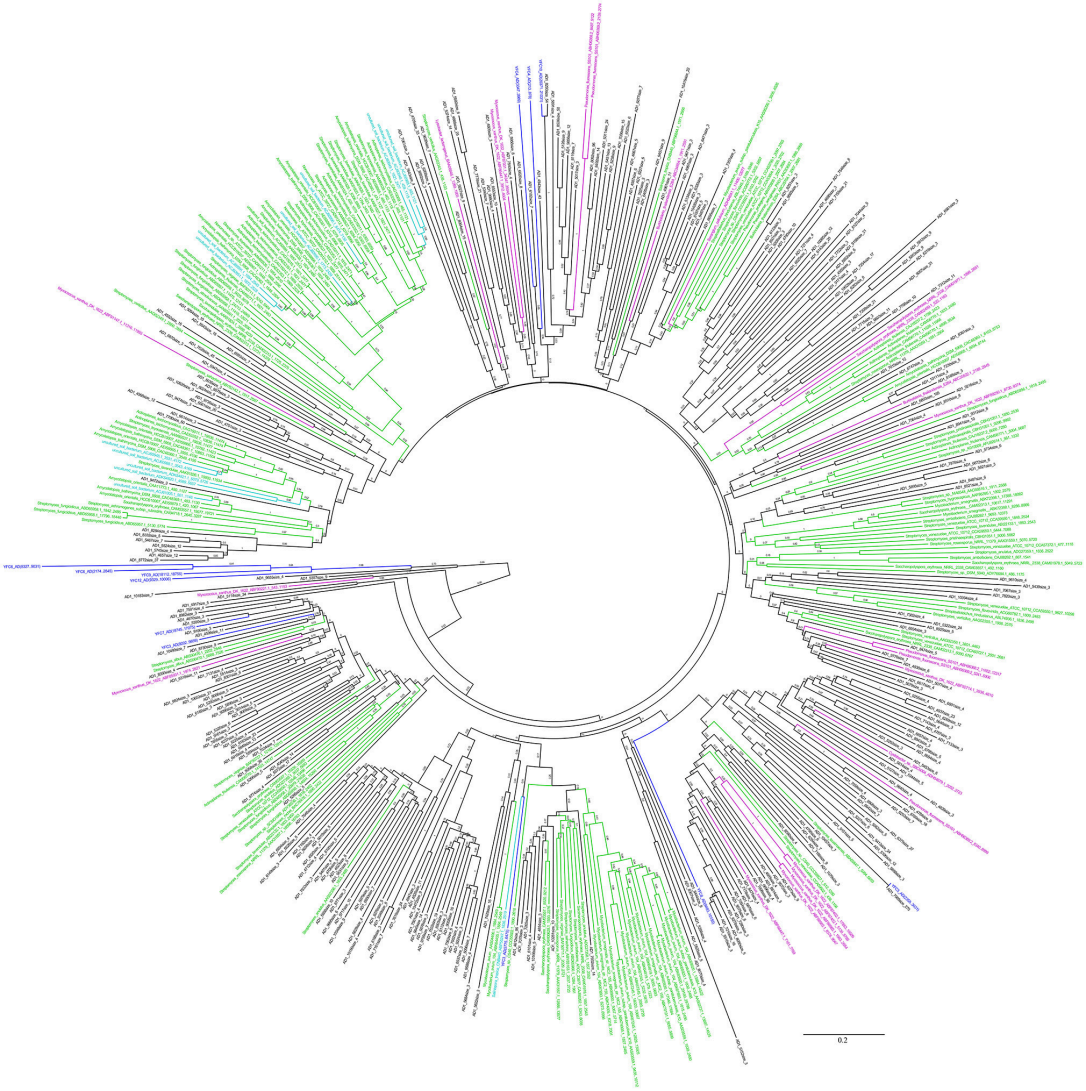
Phylogenetic tree of AD representative sequences of each OTU obtained from Yellow Sea sediment and the reference functional characterized AD sequences. All the representative sequences of each OTU clustered at 90% identities were marked with black. All the sequences identified in fosmid contigs were marked with blue. The functionally characterized AD of *Actinobacteria* retrieved from ClusterMine360 database were marked with green. The functionally characterized AD of non-*Actinobacteria* bacteria retrieved from ClusterMine360 database were marked with pink. The functionally characterized AD of uncultured bacteria retrieved from ClusterMine360 database were marked with cyan.

**Figure 4:**
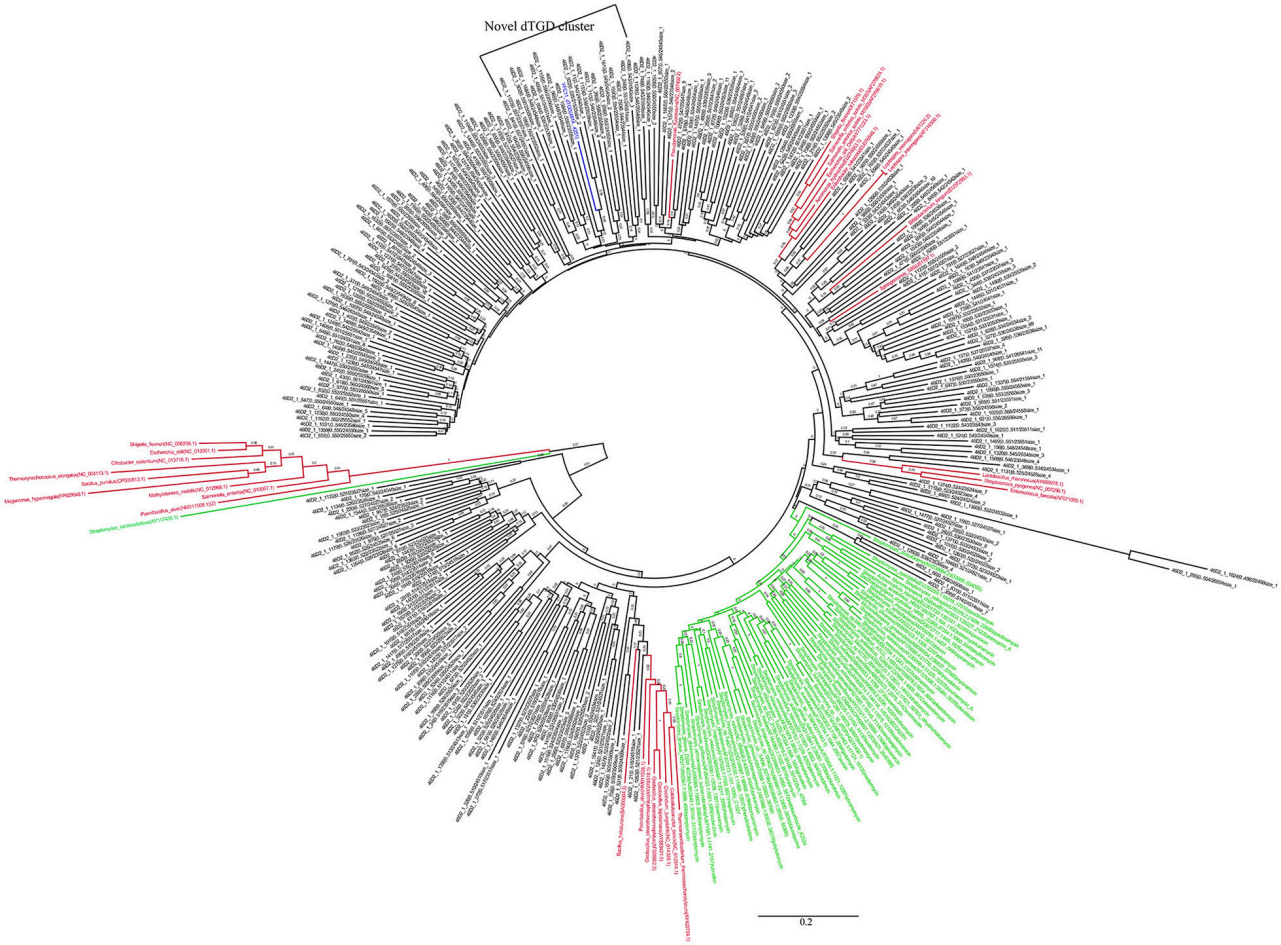
Phylogenetic tree of dTGD representative sequences of each OTU obtained from Yellow Sea sediment and the reference functional characterized sequences. All the representative sequences of each OTU clustered at 80% identities were marked with black. All the sequences identified in fosmid contigs were marked with blue. The functionally characterized dTGD identified for natural product biosynthesis were marked with green. The reference sequence of dTGD identified for primary metabolism were marked with red.

The KS and AD gene fragments were clustered with their corresponding known reference gene fragments in the phylogenetic tree, however, all of them showed less than 80% identities with the reference KS genes, suggesting they were part of novel PKS gene cluster (Figures 2, 3). In the phylogenetic trees, 7 KS representative reads (A total of 27 representative KS reads in the tree) and 66 AD representative reads (A total of 308 representative AD reads in the tree) were clustered with corresponding reference gene fragments of Actinobacteria, showing they were some potential natural product biosynthetic gene clusters of Actinobacteria bacteria (Figures 2, 3). Only few dTGD reads were clustered with known reference Actinobacteria dTGD gene fragments, suggesting that most dTGD genes of the marine sediment sample might not derive from Actinobacteria and were different from the known ones (Figure 4). Further analyses of the KS gene fragment evolutionary lineage suggested that 11 KS representative reads were clustered with known AT-less type I PKS KSs and the other 16 KS representative reads were clustered with known canonical type I PKS KSs (Figure S2).

### Screening and sequencing of fosmid clones harboring KS or AD domains

The primers of KSLF/R and A3/A7R were used to screen fomsid clones harboring natural product biosynthetic genes from the constructed fosmid clone library. Similar to the diversity analysis, AD domains were more abundant than KS domains. A total of 43 PCR-positive 384-well plates containing AD genes and 28 PCR-positive 384-well plates containing KS gene fragments were identified from all the 188 384-well plates.

For AD domains were more abundant than KS domains, eight AD gene-containing and one KS gene-containing fosmid clones were selected for further sequencing to reveal natural product biosynthetic gene clusters in the marine sediment. Due to repetitive sequences in 3 fosmid clones, a total of 12 contigs named YFC1 to YFC12 were obtained from these 9 fosmid clones (Table 4). PhyloPythiaS analysis of these 12 contigs showed that five fosmid clones were assigned to Actinobacteria, three fosmid clones were assigned to Proteobacteria and one fosmid clone was assigned to unknown bacteria (Table 4; Patil et al., 2012).

**Table 4:** Twelve fosmid contigs assembled from 9 fosmid clones harboring KS or AD gene fragments.

**Fosmid contigs ID**	**Sequences used for assembly**	**Length (bp)**	**Natural product biosynthetic domains identified**	**Taxonomic binned by PhyloPythiaS**	**Similar known gene cluster predicted by antiSMASH**
YFC1	Fosmid45M19	40,128	KS	Syntrophobacterales	Ajudazol_biosynthetic_gene_cluster (53% of genes show similarity)
YFC2	Fosmid60N12	33,750	AD and KS	Actinobacteria (class)	N/A
YFC3	Fosmid78P12	32,837	AD	Coriobacteriaceae	Gramicidin_biosynthetic_gene_cluster (16% of genes show similarity)
YFC4	Fosmid164P7	32,576	AD	Coriobacteriaceae	Jagaricin_biosynthetic_gene_cluster (13% of genes show similarity)
YFC5	Fosmid79B24R	5,655		Actinomycetales	N/A
YFC6	Fosmid65F11F	9,478	AD and KS	Actinobacteria (class)	Microsclerodermins_biosynthetic_gene_cluster (14% of genes show similarity)
YFC7	Fosmid65F11R	28,082	AD and KS	Actinobacteria (class)	Pellasoren_biosynthetic_gene_cluster (25% of genes show similarity)
YFC8	Fosmid60H14	27,713	AD	Bacteria	N/A
YFC9	Fosmid79B24F	27,636	AD and KS	Actinobacteria (class)	N/A
YFC10	Fosmid163L15	26,927	AD	Deltaproteobacteria	N/A
YFC11	Fosmid8H1R	24,345	AD	Proteobacteria	Sphingan_polysaccharide_biosynthetic_gene_cluster (13% of genes show similarity)
YFC12	Fosmid8H1F	13,106	AD	Oceanospirillales	N/A

*N/A shows no identity with known natural product biosynthetic gene clusters*.

### Natural product biosynthetic gene analysis of the contigs

**Figure 5:**
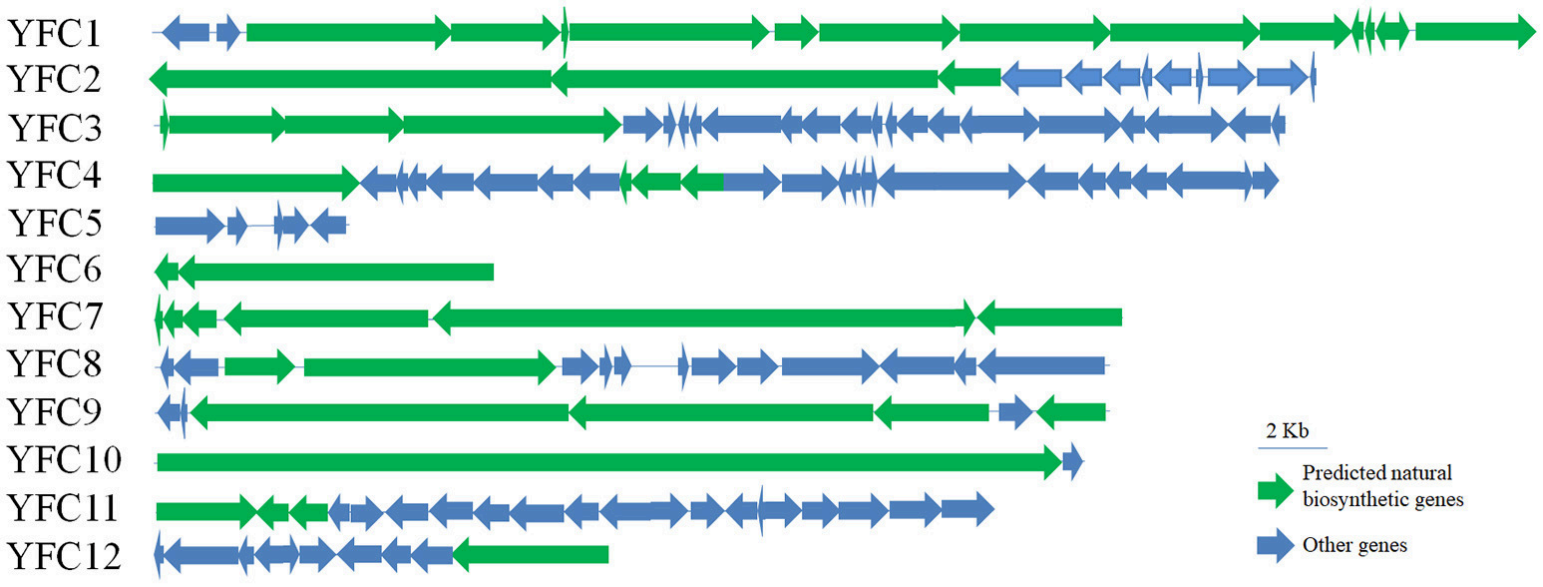
Gene organization of the 12 fosmid contigs harboring natural product biosynthetic gene clusters were shown. The scale bar of 2 kb was marked.

Annotation of the 12 contigs showed that 11 contigs contained natural product biosynthetic gene clusters, among which, four contigs harbored PKS/NRPS hybrids gene clusters, one contig harbored PKS gene clusters, and six contigs harbored NRPS gene clusters (Table 4 and Figure 5). Especially, one glycosyl transferase gene clustered with natural product biosynthetic genes was identified in contig YFC3 and one dTGD gene fragment YFC_dTGD(3787…4830) was identified in YFC11 (Figures S1A,C). Moreover, two contigs of YFC8 and YFC11 might have full-length NRPS gene clusters, because genes of natural product biosynthetic pathway usually clustered in the genome and there are some nonnatural product biosynthetic genes flanking around the NRPS gene clusters. Some of another nine contigs were predicted to harbor several completed PKS or NRPS modules, but not completed natural product gene clusters (Figures S1B,C). All the PKS or PKS/NRPS hydrids contigs harbored AT domains in their PKS modules, suggesting these contigs harbored canonical type I PKSs (Figure S2).

Six KS gene fragments, 12 AD gene fragments and one dTGD gene fragment were identified in the 11 contigs using the primer-mapping search. Among them, one AD gene fragment YFC3_AD(2358…3031) was the same as the most abundant AD gene fragment (AD1_7989size_270) identified by 454 pyrosequencing (Figure 3), but other AD, KS and dTGD gene fragments identified in the contigs were different from the corresponding ones obtained by 454 pyrosequencing. The dTGD genes in YFC11 (YFC11_dTGD) were clustered with natural product biosynthetic genes, suggesting it might be one dTGD gene used for natural product glycosylation. The cluster harboring YFC11_dTGD and other obtained dTGD genes were different from the known dTGD genes, suggesting this cluster might represent one novel dTGD clade used for natural product glycosylation (Figure 4). Besides, five KS gene fragments of YFC1 clustered together in the phylogenetic tree (Figure 2). In the meanwhile, some AD gene fragments derived from same contigs were not clustered together in the phylogenetic tree, such as three AD gene fragments of YFC3_AD(2358…3031), YFC3_AD(5775…6470) and YFC3_AD(9202…9859) distributed in 3 different clades. Moreover, all the 6 identified KS gene fragments were clustered with canonical type I PKS KS domains, further showing that the contigs of YFC1 and YFC2 harbored canonical type I PKSs (Figure S2).

## Discussion

In this study, the 16S rRNA gene analysis indicated that *Proteobacteria* was the most dominant phylum in the Yellow Sea sediment, which was similar with bacteria diversity distributed in the East China Sea (south of the Yellow Sea) and the South China Sea (south of the East China Sea) where *Proteobacteria* was the most dominant phylum (Lu et al., 2011; Zhu et al., 2013). NRPS and PKS gene clusters are widely distributed in *Proteobacteria* (Wang et al., 2014). Moreover, 25% of the tested bacteria of the East China Sea possessed biological activities and some of them can produce novel natural product compounds (Lu et al., 2011), hinting natural product biosynthetic gene clusters in the marine sediment would be abundant and used for novel compounds biosynthesis. Only 11 OTUs (0.8% of all the OTUs) were assigned to *Actinobacteria*, but they were different from the isolated *Actinobacteria* strains from the same sample, suggesting that metagenomic method revealed some *Actinobacteria* bacteria which were different from the isolated ones (Xiong et al., 2015). However, five of the nine recovered fosmid clones were assigned to *Actinobacteria*, this may due to the fact that A3F/A7R and KSLF/R primers were designed with known *Actinobacteria* genes and it was easier to obtain *Actinobacteria* KS and AD genes than other bacteria KS and AD genes (Ginolhac et al., 2004; Ayuso-Sacido and Genilloud, 2005). Moreover, three of the sequenced nine fosmid clones were assigned to *Proteobacteria*, further showing *Proteobacteria* was the most abundant bacteria and was the potential natural product biosynthetic producers in the Yellow Sea sediment.

Computationally screening of the NCBI-NT database with the primers KSα F/R showed that all of the identified KSα genes were from phylum *Actinobacteria* (Reddy et al., 2012). Though different PCR conditions had been tried, no KSα gene fragments were amplified from the marine sediment, the reason might be that *Actinobacteria* bacteria were rare in Yellow Sea sediment and few PKS-II KSα domains distributed in this sample. More than half of the AD and KS genes in Uniprot database were from *Actinobateria* (Minowa et al., 2007; Reddy et al., 2012). Only part of the AD representative reads of each OTU were clustered with known *Actinobacteria*-derived reference genes and *Actinobacteria* represents only a small proportion of all the bacteria in the Yellow Sea sediment, suggesting some NRPS gene clusters which were different from the known ones distributed in the samples. The most abundant AD read was the same as one AD gene identified in the fosmid contigs, showing AD gene fragment diversity evaluated by PCR amplification might cover most of the abundant NRPS in the samples. Due to the fact that no corresponding KS reads were same with the KS genes identified in the fosmid contigs, hinting PCR amplification bias introduced by the primers or other factors may lead us to underestimate the true diversity of PKS-I in the marine sediment. Besides, more than one KS or AD domains were distributed in one natural product biosynthetic gene clusters, such as YFC3 contains 3 AD domains (Figure 3), suggesting metagenomic sequencing of the microbial community should be tried to help evaluate KS or AD diversity.

The recovered high dTGD diversity and most representative dTGD reads were not clustered with the reference dTGD genes, suggesting bacteria in the marine sediment might encode diverse natural products in the Yellow Sea sediment tend to be glycosylated and there would be some novel 6DOH-modified natural products (Figure 4). One dTGD gene clustered with predicted natural product biosynthetic genes identified in YFC11 showed only 67.2% amino acid identity with its nearest known dTGD genes, further implying that dTGD in the Yellow Sea had potential to help synthesize novel natural products (Figure 4). Moreover, one glycosyl transferase (GT) gene was identified to be clustered with one natural product biosynthetic gene cluster, suggesting there might be some glycosylated natural products in the Yellow Sea sediment.

Most natural product biosynthetic genes in the recovered fosmid clones showed low identities with the known natural product biosynthetic genes clusters (Table 4), suggesting there might be abundant novel natural product biosynthetic gene clusters in marine sediment of the Yellow Sea. Though only two potential natural product gene clusters were completely recovered, these gene clusters give us insights into the potential natural products of the Yellow Sea sediment (Figure S3). Moreover, AT-less and canonical type I PKS KS gene fragments were identified in the metagenomics analysis (Figure S2), suggesting different kinds of PKs were available in the marine sediment. With the development of synthetic biology, rational design of natural product biosynthetic gene clusters with recovered natural product gene modules in this study and express them in appropriate chassis cells would be promising way to produce novel natural products (Menzella et al., 2005; Winter and Tang, 2012; Cobb et al., 2013; Montiel et al., 2015).

The bacteria community in the marine sediment of the Yellow Sea is highly diverse and the natural product biosynthetic genes are different from the ones identified in soils (Reddy et al., 2012), suggesting the ocean might be a rich source for novel natural products discovery. Moreover, our study suggests that the culture independent metagenomic method not only shows bacterial and natural product diversity of the Yellow Sea sediment, but also helps reveal novel PKS and NRPS gene clusters.

## Author contributions

Conception and design: XY, ZZ; Data acquisition: YW, LZ, XY; Analysis and interpretation of the data: YW, LZ; Drafting of the article: YW, LZ; Critical revision of the manuscript: XY, ZZ.

### Conflict of interest statement

The authors declare that the research was conducted in the absence of any commercial or financial relationships that could be construed as a potential conflict of interest.
