# Supplementary material for: Diversity of Gene Clusters for Polyketide and Nonribosomal Peptide Biosynthesis Revealed by Metagenomic Analysis of the Yellow Sea Sediment
Source: Front Microbiol. 2018 Feb 27;9:295. doi: 10.3389/fmicb.2018.00295 (PMC5835077; doi:10.3389/fmicb.2018.00295)
Supplement: Supplementary file 1 [file Presentation1.pdf]

Diversity of gene clusters for polyketide and nonribosomal peptide biosynthesis revealed by metagenomic analysis of the Yellow Sea sediment

Running title: Natural product gene diversity of marine sediment

Yongjun Wei[1], Lei Zhang[1], Zhihua Zhou[1], Xing Yan[1]

[1]CAS-Key Laboratory of Synthetic Biology, Institute of Plant Physiology and Ecology, Shanghai Institutes for Biological Sciences, Chinese Academy of Sciences, Shanghai 200032, China.

Correspondence

Dr. Xing Yan

E-mail: yanxing@sibs.ac.cn

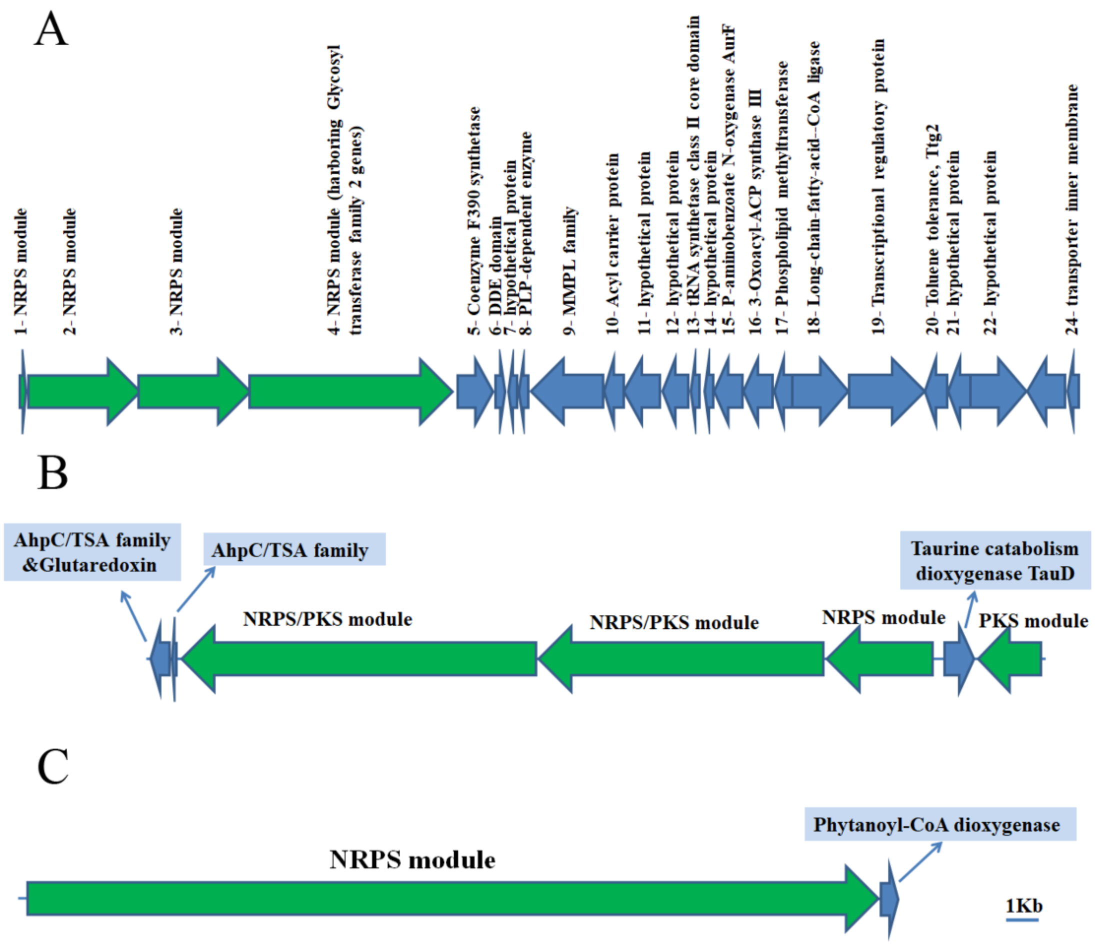

Supplementary Fig. S1 Gene organizations of 3 contigs YFC3 (A), YFC10 (B) and YFC5 (C) recovered from Yellow Sea sediment. The fosmid contigs were annotated with FGENESB (http://linux1.softberry.com/berry.phtml) and BLASTX.

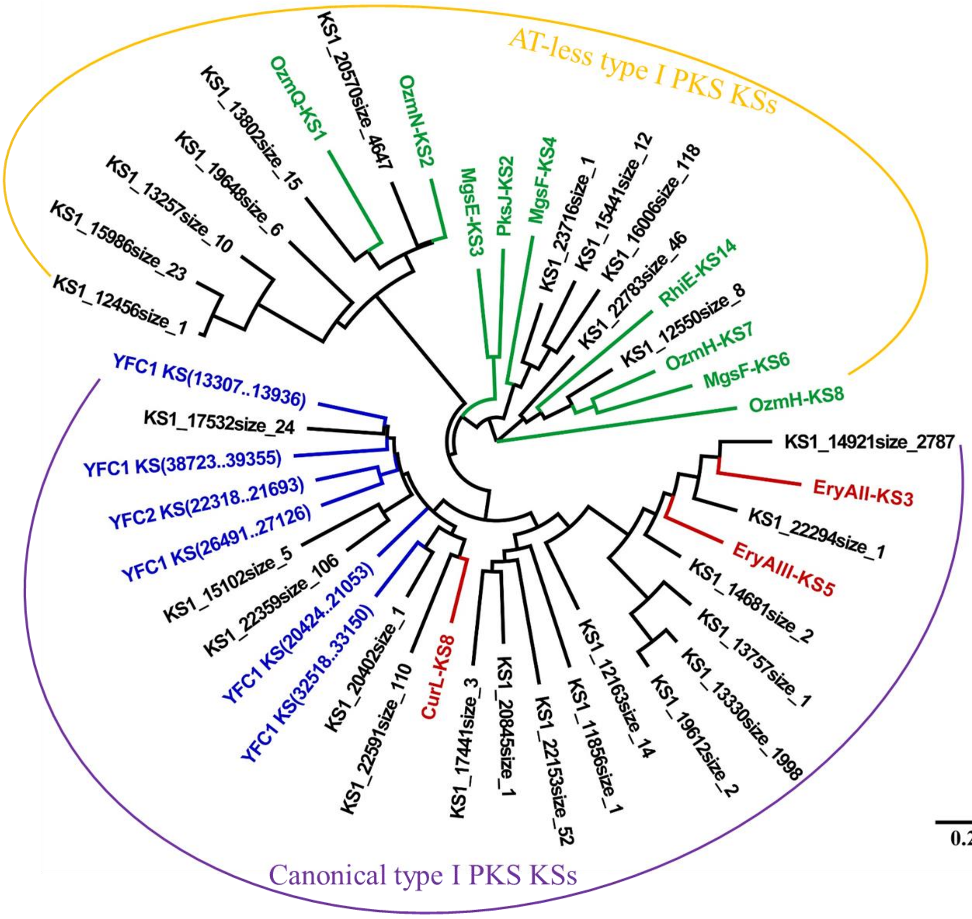

Supplementary Fig. S2 Phylogenetic ~~trees~~ of KS representative sequences of each OTU obtained from Yellow Sea sediment and the refenece functional characterized sequences. The representative KS sequences of each OTU were marked with black; The sequences identified in fosmid contigs were marked with blue; The reference AT-less type I PKS KSs were marked with green; The reference Canonical type I PKS KSs were marked with green.

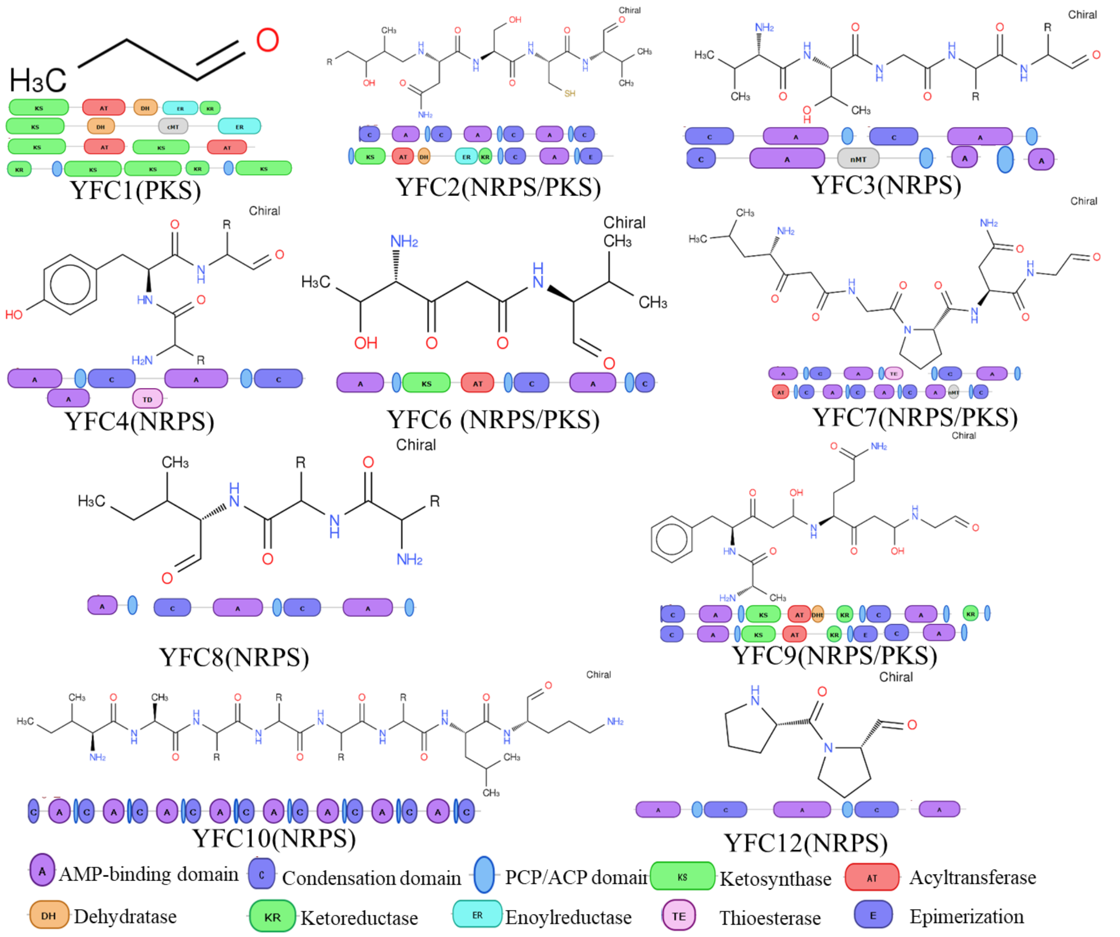

Supplementary Fig. S3 Potential PKS or NRPS substructure biosynthesized by the fosmid contigs predicted with antiSMASH were shown. All the NRPS/PKS domains of each contig used to predict the structure were shown underneath the structure. Rough prediction of core scaffold based on assumed PKS/NRPS colinearity; tailoring reactions not taken into account.
